# Friendship groups and physical activity: qualitative findings on how physical activity is initiated and maintained among 10–11 year old children

**DOI:** 10.1186/1479-5868-6-4

**Published:** 2009-01-12

**Authors:** Russell Jago, Rowan Brockman, Kenneth R Fox, Kim Cartwright, Angie S Page, Janice L Thompson

**Affiliations:** 1 Department of Exercise, Nutrition & Health Sciences, University of Bristol, Bristol, UK; 2 School of Psychology, University of Southampton, Southampton, UK

## Abstract

**Background:**

Many youth physical activity interventions have minimal effect. To design better interventions we need to understand more about the factors that influence youth activity. Application of self-determination theory to youth physical activity, particularly the relatedness and competence, might suggest that friends and friendship groups influence the initiation and maintenance of youth physical activity. In this study we examined this issue.

**Methods:**

Seventeen focus groups were conducted with 113, 10–11 year old children, from 11 primary schools in Bristol, UK. Focus groups examined: 1) the nature of children's friendship groups; 2) associations between physical activity and social group status; and 3) how friendship groups affect the initiation and maintenance of physical activity. All focus groups were audio-taped and transcribed verbatim. Data were analyzed using content analysis.

**Results:**

Participants reported that there were three different types of friendship groups; School friends; Neighborhood friends; and Other Friends who were friends from organized activities or children of their parents' friends. Participants had multiple groups of friends and engaged in different activities with the different groups. Possessing several groups of friends was desirable as it kept the friendships fresh and interesting. Physical activity was perceived as a positive attribute and linked to social status among boys. Among girls the association between physical activity ability and social status was more complex, appearing to differ by the norms of the group to which participants belonged. Some participants reported that low activity ability could be perceived as desirable in some social groups. Participants reported that friends provide support to initiate physical activity via co-participation (i.e. engaging in activity together); modeling of being active; and providing verbal support to engage in activity. Enjoyment was the most important factor in maintaining activity participation with participating in activity with friends a key factor influencing enjoyment.

**Conclusion:**

Friendship groups affect both the initiation and maintenance of youth physical activity. Children belong to several groups and engage in different activities with different groups. Simple strategies that aim to promote physical activity via the different friendship groups could be an effective means of promoting increased physical activity in young people.

## Background

Physical activity has been associated with lower insulin [1,2] and glucose [1,2] levels among children and adolescents [3]. Physical activity is also associated with improved emotional well-being and self-esteem among youth [4,5] and provides youth with opportunities to learn new social skills [6] and enhance personal development [7]. Unfortunately, despite the benefits of regular physical activity, many youth [8-10] do not meet the current UK recommendation of an hour per day of physical activity on most days of the week [11,12]. Physical activity levels decline during childhood, with the start of secondary school (11 years of age) identified as a key period of change [13,14]. The risk of overweight children becoming overweight adults increases from an odds ratio of 10.3 at ages 6–9 to 28.3 at 10–14 [15]. Thus, preventing a decline in physical activity at the start of secondary school is likely to make an important contribution to the prevention of obesity.

Recent review articles have reported that the majority of youth physical activity [16] or youth obesity prevention programs with a strong emphasis on increasing physical activity [17,18] have reported no, weak or minimal effects on obesity or physical activity. The mediating variable model [19] suggests that change in physical activity behavior will be achieved by understanding the key factors (mediators) of the behavior. Self-determination theory (SDT) [20] has been used to understand adult physical activity [21] and is being applied to understand youth physical activity [22]. According to SDT, individuals have three innate psychological needs: competence, relatedness and autonomy that affect behaviour and behaviour being either intrinsically motivated, extrinsically motivated or amotivated with intrinsically motivation desirable for the long-term adoption of a behaviour [20]. Friends are likely to be important to youth activity because a child who feels confident (competence) that he/she can be active in front of his or friends is perhaps likely to be more active. Similarly, physical activity with friends is likely to build a sense of relatedness and autonomy to engage in independent physical activity. Support for the importance of friends can be drawn from the data which shows that engaging in physical activity with friends has been associated with increased motivation for being active [23] and more intense physical activity among youth [24].

Although SDT would suggest that friends and friendship groups are likely to be key influences on youth activity the construction of friendship groups and friends' potential influence on both the initiation and maintenance of youth physical activity is not clear. Moreover, although sporting ability has been linked to social status and popularity among some youth [25], it is not clear if these associations are uniform for all youth or if they differ by gender. Further examination of the link between physical activity ability, social status and friendship groups may provide insights into how key group attitudes are formed and how they may impact on current and future physical activity participation. We employed qualitative methods to addresses these research gaps among 10–11 year old children by examining: 1) the nature of children's friendship groups and if children engage in different activities in different settings; 2) associations between physical activity and friendship group status and how this might be harnessed to promote physical activity; and 3) how friendship groups affect the initiation and maintenance of physical activity.

## Methods

Participants were 113, 10–11 year old children recruited from 11 primary schools in Bristol, UK. The schools were recruited to approximate the economic diversity of the local area based on the Index of Multiple Deprivation (IMD), a UK government produced area level measure of deprivation that includes assessments of income, employment, health and education [26]. We obtained the IMDs for the postcodes of all local schools and then recruited 4 schools from the lowest third (Low SES schools), 4 from the middle third (Middle SES schools) and three from the highest third (High SES schools). Although this approach only provides information on the school and not where the children live it was intended to provide a reasonable range of participants from different economic neighborhoods within the city. Once schools were recruited we held a briefing session for all Year 6 students (10–11 years of age) in the school in which we invited volunteers to participate in a focus group to tell us about physical activity and their friends. One or two focus groups were then held at each school between March and June 2007 with 2–12 participants per group. The study was approved by the School of Applied Community and Health Studies Ethics committee at the University of Bristol (ref 017/06) and informed parental consent and childhood assent was obtained for all participants [27].

Focus groups were selected as the data collection format. Focus groups facilitate the collection of personal thoughts about the topics being discussed in an environment which is perceived to be safe [28]. Focus groups have been identified as a particularly useful means of obtaining new data from children [29] and are an effective method of getting information about attitudes and opinions on topics of interest. Each focus group lasted 30–45 minutes and was conducted by a trained moderator. All focus groups were recorded using an Olympus DS-2200 digital recorder and an assistant moderator took notes of salient events that occurred during the session. The focus groups had a semi-structured design with follow-up probes on key topics of interest. Questions were piloted in one school with a group of 10–11 year old students prior to data collection. Questions focused on the types of activities in which the children engaged and with whom; the association between sporting ability or physical prowess and social status within the peer group; and the ways in which peers encourage physical activity participation particularly in relation to the initiation and maintenance of behaviors.

### Analysis

All recordings were transcribed verbatim and anonymized. A second researcher listened to the recordings and checked the transcripts for accuracy with any differences reconciled by a third researcher. Consistent with content analysis [30], transcripts were read line by line and marked with independent codes that described the content of the response. Codes were entered as free nodes (i.e. labels that described themes) into a newly created database in NVivo (Version 2.01, QSR, Southport UK). Codes were checked by a second investigator and matrices of codes were developed and hierarchical codes (categories that described a broader group of themes) produced. Text retrievals were then performed on hierarchical codes and contents were interpreted and summarized into tables.

## Results

Participant characteristics are shown in Table 1. Seventeen focus groups were conducted with 113 participants from 11 schools with the sample being 52% female. Individual economic, physical activity and body mass data were not measured in this study.

**Table 1:** Participant characteristics

**School SES**	**# Schools**	**# Focus Groups**	**N**	**Boys n (%)**	**Girls n (%)**
Low	4	5	27	15 (55.5)	12 (44.5)

Middle	4	6	41	17 (41.5)	24 (58.5)

High	3	6	45	22 (48.9)	23 (51.1)

**Total**	**11**	**17**	**113**	**54 (47.8)**	**59 (52.2)**

### Friendship groups

Three broad types of friendship groups were described by participants: 1) School friends; 2) Neighborhood friends and; 3) Other friends. A summary of the three groups is presented in Table 2 and discussed below.

**Table 2:** Friendship groups reported by 10–11 year old youth

**Group**	**Characteristics of the group**
School friends	• Friends from school.
	• Specific groups are formed at the school site by children, with activity outside of school arranged at school or by the child's parents.

Neighborhood friends	• Friends who reside close the child's house.
	• Groups are often self-forming and very independent.

Other friends	• Friends from activity clubs such as football team or swimming club.
	• Children from other non-activity clubs such as scouts, guides or a local community group.
	• Children of the child's parents' friends.

#### School friends

Most participants reported that they had strong friendships with children from school with whom they spent considerable time outside of school.

*"... school friends, we go almost everywhere I go" *(Female, Middle SES *school*).

*"...my friends from school we play football, cos that's the only thing we play" *(Male Low SES school).

#### Neighborhood friends

Many participants and particularly those who attend schools in lower socio-economic areas reported that they spent a lot of their time outside of school with friends from the local neighborhood.

*" Um.. well usually on the weekend I just go out on my bike and ride around with my friends" *(Male, Low SES school).

*"... the friends that live in my street I normally hang around with*..." (Male, middle SES)

#### Other friends

Participants reported that they have other friends from outside of school. These friends were located at organized activity groups such as football club, swimming or youth activities such as scouts and guides and family friends.

*.."friends I have known for a while and mum and dad's friends, things like that." *(Male, Middle SES school)

*"Yeah, football club, all of them go to football club ...um and meet people through my mum's friend's sons something like that" *(Male, Low SES school)

*"I've got a few at school *[friends), *I've got some other friends like my parents' friends with then and then, they've got children so I'm friends with them" *(Female, High SES School).

*"Well I know a group in school and when I go to my Dad's I see another group of friends" *(Female, Low SES School)

It is important to highlight that the majority of participants reported spending time outside of school with more than one type of friend and often the activities in which they engaged were different for different types of friend. For example,

*"I have 3 groups really, I have my school friends, people I play rugby with and my old school friends because I changed school in year 3" (*Male, High SES School)

*"...in school we like play more but when we're out of school just like riding the bike and stuff" *(Female, Low SES School).

*"I play with my rugby friends at rugby, but I don't really play with them anywhere else because they live, most of them live quite a long way away, with my school friends I usually just like hang around with them round my house and their house, playing football, sometimes or cricket" *(Male, High SES School)

Participants also reported that having different groups of friends and engaging in different activities with each group was desirable as it kept the activities interesting.

*"If you have the same group of friends all the time it gets boring, because they like do the same things like, say it would be a bit boring if me and [name of another child] hang about all the time cos it would be just like football..." *(Male, Middle SES School)

*"Well, um, people from scouts I just see them on Tuesdays I don't really like, like go, out with them so, so I, yeah I just usually see them at scouts and just...they're like, Goths or Emos and stuff like that, so it's like, we do weird things..." *(Female, Middle SES School)

*"...you get to know people and their different personalities and stuff and you get to...enjoy like different activities that they all like in a group, like you get to kind of know more people "" *(Female, Middle SES School)

*"...it's fun to have a variety of friends and a variety of friends' interests, so you get to, do more things and do different things" (*Male, Middle SES School)

*"Well all the different groups of friends that I have they are all different so its kind of you get a different variety of friends and different kinds of people you get to know and so it's kind of like sharing all the different sides of you that you have*..." (Female, High SES School)

### Associations between physical activity and social group status

Boys and girls reported different associations between physical activity ability and social group status. Gender specific quotes are presented below and the theme is summarized in Table 3.

**Table 3:** 10–11 year old children's reports of the associations between physical activity and social status, and how friends help to initiate and maintain physical activity participation

**Theme**	**Summary of key items within the theme**
Associations between physical activity and social group status	• Being good at sports (particularly football or "soccer") is a key status symbol for boys; boys popular at sports also seems to be a key factor in identifying leaders within the school.
	• For girls the link between physical activity and social status is less clear. For some girls being good at sports is seen as a positive status symbol, in other group poor sporting ability also has high social status.
	• The perceived gender difference in this perception was reported by both boys but participants from lower SES schools seemed to be able to articulate this difference more clearly.

Initiation of physical activity	Friends provide social support to start physical activity via:
	• Co-participation in physical activity, for example by a friend taking the child to the new activity.
	• Modeling of being activity by older children or friends
	• Verbal support to initiate a new physical activity; particularly in the form of joining new activity clubs.

Maintenance of physical activity	The maintenance of active behaviors was influenced by two factors:
	• Physical activity is enjoyable
	• Comparison of active and sedentary pursuits: Participants commented that the social aspects of activity are often preferable to the more solitary pursuits that can be associated with screen-time.

Physical activity ability was perceived by almost all boys as a desirable status symbol, with increased ability being associated with popularity and peer leadership. Sample quotes are presented below

"... *with the boys it is because all the people that are good at football, they all like them*" (Male, Low SES School).

"...*Um, you have to be quite good at football and stuff, cos I think most people like really like football and they expect you to be quite good at it, yeah*" (Male, Middle SES School).

*"I would say I think it helps your popularity in the boys' group to be physically active*..." (Female, Low SES School)

The association between physical activity and peer group status was less clear among the girls. For example, among some girl peer groups physical activity ability was perceived as a good indicator of social status and it appeared that for these girls friendship groups were formed around physical activity groups.

" *..Everyone wants friends that are sporty so if you are sporty you become friends with people who are sporty*.." (Female, High SES School).

" ...*she's most popular because she's in this football club but she plays for the 10s and 11s and the Under 14s*..." (Male, Low SES School).

However, some girls reported that physical activity ability could be seen as a negative attribute among peers and that other aspects of life were perhaps more important. For these girls group affiliation was based upon non-physical activity behaviors and poor activity ability appeared to be both the group norm and essential for group membership.

"*I think in our group if you are absolutely hopeless at sports it makes you more popular*" (Female, Low SES School).

*" I think [name]'s quite popular, and I think it's cos, I don't know she goes shopping and gets all these new clothes and, she's like um, she likes talking to the boys and things which somehow makes her popular" *(Female, Middle SES School)

Interestingly, the gender difference between physical activity and peer status was reported by boys and girls, suggesting that where physical activity engagement is seen as a negative attribute among the girls this perception is also acknowledged by the girls. For example:

"..*by the looks of it boys seem to judge people by the way that they are by their *[sport]*skills and how they act, the girls seem to judge on looks and how they are*" (Female, Low SES School).

"*..girls are popular with each other cos they've got all fancy little make-up things, boys are popular because they like football*" (Male, Low SES School).

It is important to highlight that in analyzing this section of the data it became apparent that participants from lower SES schools found it easier to articulate how physical activity was or was not a status symbol and particularly the gender differences. This difference appears to suggest that participants from lower SES schools were more aware of social roles and gender differences than their counterparts from higher SES schools.

### Initiation of physical activity

Many participants reported that their friends had helped them to initiate physical activity and that this initiation occurred via one or more of three mechanisms: 1) co-participation with a peer who was also already engaged in the activity; 2) modeling by a friend who already engaged in the activity; and 3) verbal encouragement. Each mechanism is illustrated below.

#### Co-participation in physical activity

"...*because my friends were doing it and they just took me along and I watched it one lesson and then the next I wanted to have a go*." (Female, Low SES School).

"*..I wanted to do them [activity] and also it encouraged me because lots of my friends wanted to do it as well*." (Male, High SES School).

"*my friend [child's name] encouraged me to go to cubs because she said she was going and it was really fun*..." (Female, Middle SES School)

#### Modeling of being active

"*last year when the Year 6s were here like they were like doing really well and really enjoying it and like doing lots playing lots of matches and then like my friends [name] and [name] and well my friends yeah said 'oh shall we have a go' and I'm like, I wasn't quite to start off with I wasn't quite sure if that was the right sport for me, but I just thought maybe I could give it a go *..." (Female, Low SES School)

"*Well first of all, all of my friends were involved in it and it looked quite fun*" (Female, Low SES School)

"*if a friend does it [activity] you wanna do it and um, it will make it a bit more enjoyable, cos...if you like have to do like the sport but you don't really like that sort of sport, if your friend's doing it you can have a laugh while you're doing it so you forget that you don't like that sport, you just get on with it" *(Female, Middle SES School)

#### Verbal support to initiate a new activity

Participants reported that social support was provided via verbal encouragement, particularly in relation to joining new organized physical activity opportunities with a peer actively encouraging them to engage in physical activity.

" ...*my friend *[child's name]*encouraged me to go to cubs [Junior Scouts] because she said she was going and it was really fun*." (Female Low SES School).

"...*with the cricket my friends encouraged me because at school all of them ... and in my first football club my friend encouraged me *[to go]" (Male, Middle SES School).

### Maintenance of physical activity

Participants reported that two factors influenced whether they maintained their participation in physical activity, the enjoyment of the activity and the perception that active pursuits were preferable to sedentary ones. Each of these themes is presented below.

#### Enjoyment of physical activity

Enjoyment was reported by the majority of the participants as being central to regular attendance in physical activity with many participants commenting on the intrinsic appeal of physical activity.

"*..it's fun playing with your friends*" (Female, High SES School)

"*I like doing dancing cos it's just fun*" (Female, Middle SES School)

"*I like basketball because I like the sport*" (Male, Low SES School)

Many participants reported that they took part in physical activity for enjoyment or because of the perceived "fun" and the social aspects of activity. However, when probed as to why they found activity enjoyable friendships or social aspects of physical activity were often offered as a common reason for enjoyment and no other reason for the perceived enjoyment was articulated.

"*Um, because you get to spend time with your friends [pause] and that's it really*" (Female, Middle SES School)

"you do, you usually do it because you want to hang around with your mates and then also, sometimes you find it more interesting because you're there with your mates" (Male, High SES School)

"I like doing lots of sport, and yeah I probably prefer to be with my friends and play a sport" (Male, Middle SES)

### Comparison of active and sedentary pursuits

Participants also highlighted that being engaged in physical activity with friends was often seen as more preferable than engaging in less active, more solitary pursuits such as the screen-viewing behaviors of TV watching or computer use.

"*...I mean, if you stay at the house just doing nothing all the time and just going on like the computer.. you'll.. just come home and just do it again but you need to actually get out and do something with your friends.. it keeps you active and you can have fun*" (Male, Low SES School).

"*..If somebody said 'play' to me I would think of fun and playing sports I wouldn't really think of playing anything else but sports, I wouldn't want to go on my computer or anything*" (Male, Middle SES School).

## Discussion

The data presented here indicate that friendship groups are key influences on the location and type of physical activity in which children engage. Friendship groups are not uniform and differ for individual children with many children belonging to several groups and engaging in different activities with the different groups. Participants reported that membership of several groups was desirable as it presented alternative opportunities for activity and prevented boredom. Diverse friendship groups may therefore be a mediator of higher levels of physical activity because if a child has more friendship resources to draw upon he or she may be more active. Moreover, if one friendship group dissolves the child can still be active with other groups, thereby providing a "safety net" against inactivity. Finding ways to help children to develop physical activity behaviors with several different groups could be a key method for families and policy makers to increase youth physical activity.

The potential utility of membership of several friendship groups raises a number of issues about current youth physical activity research. A large proportion of youth physical activity research is conducted within schools and is based on the underlying premise that the factors that affect physical activity will be based upon either key psychosocial constructs [31], parental or home factors [32,33] or more recently the physical environment [34] in which the child resides. Studies do not focus on the extent to which friends from the neighborhood, children of parental friends or peers from organized sport or non-sport locations influence physical activity. It seems plausible that a greater understanding of the factors that influence youth physical activity participation will be achieved by identifying these groups and their mechanisms of influence on youth physical activity. To achieve this objective there is a need to develop new measures of assessing friendship group affiliation and specifically if membership of several friendship groups results in higher levels of physical activity.

The results presented here suggest that children are initiated into physical activity via co-participation, peer verbal encouragement or modeling by another child. Modeling and verbal persuasion are key processes in the development of self-efficacy [35] which has consistently been related to youth physical activity levels [36,37]. Our data therefore suggest that many youth are organically increasing their own self-efficacy and thus we should explore how we can build on these processes to help youth become more active. Moreover, participants were members of several groups and thus it seems plausible that the initiation into friendship groups likely occurred by an existing friend initiating a child into a physical activity friendship group via one or more of the above strategies. Findings therefore suggest that simple strategies to build self-efficacy via co-participation, peer verbal encouragement or modeling could therefore be effective means of increasing physical activity and broadening a child's number of friendship groups. For example, encouraging children to "sign up a friend" to join them in attending an activity may spark the friend's interest in that form of physical activity. This could be achieved by providing children with "2 for the price of 1" vouchers for key physical activity venues. These strategies could easily be incorporated into physical activity interventions.

Consistent with previous research [38-40], participants reported that enjoyment of physical activity and spending time with friends were the key influences on maintaining participation in physical activity. Interestingly, our findings extend that work by indicating that physical activity is often seen as a more pleasurable way to spend time than screen-based sedentary behaviors. Therefore strategies or messages that directly contrast the perceived merits of physical activity and screen-viewing and reinforce the social aspects of physical activity could form components of strategies to maintain activity among youth. Moreover, interventions that directly focus on providing social opportunities for activity may be effective activity promotion mechanisms to harness the intrinsic enjoyment of physical activity that was expressed by these young people.

In this study we found that the association between physical activity ability and social status was complex and differed by gender. Among boys, physical activity ability was associated with a positive social status. Our findings are therefore consistent with previous work which has shown that physical activity competency is associated with both perceived and actual peer group acceptance and peer affiliation among youth [25]. For girls, our data suggest that the link between physical prowess and friendship groups is less clear, with physical activity ability being perceived either positively or negatively and this perception appearing to be related to the friendship groups to which the girls belong and the attitudes of that group. As negative childhood physical activity experiences have been shown to adversely affect adult physical activity participation [41], it seems plausible that the friendship groups to which a child belongs is likely to be a moderator of this association. Therefore, further exploration of the link between social status, physical ability and group norms among youth is needed, particularly whether using this link between physical activity and group affiliation could be a means to engage low-active boys into physical activity. Hence, we need to understand more about how allegiance to friendship groups among girls is formed and its relationship to physical activity participation among girls.

### Limitations

Although the sample in this study is relatively large for qualitative work and we attempted to recruit participants from a range of schools, it is difficult to generalize to the larger population. It is also important to recognize that as the data were collected in schools only students attending schools on data collection were able to participate. As such, the data presented here may not represent the view of children who are frequently absent from school who may have different physical activity patterns or associations with friendship groups. Moreover, no data were collected on the ethnic group of the participants which is important as the perspectives and experiences of children from different ethnic groups may vary. All qualitative work is subjective and reliant on the researchers' interpretation of the data [28] but we attempted to minimize this issue by having two researchers code the data. Moreover, the Moderator and Assistant Moderator wrote up notes from focus groups within 24 hours to prevent deterioration of any key recollections.

## Conclusion

Friendship groups exert considerable influence on youth physical activity behaviors, but these influences are complex and multifaceted. Children are often members of several friendship groups and the activities in which a child engages are influenced by the groups to which he or she is a member. Friends help to initiate children into physical activity via co-participation, modeling and verbal support and spending time with friends is an important factor in maintaining engagement in physical activity. Collectively these findings highlight that friends and friendship groups are likely to be central to the adaptation and maintenance of physical activity among youth and that activity promotion strategies that are based around friendship groups could be an effective means of engaging youth in physical activity.

## Competing interests

The authors declare that they have no competing interests.

## Authors' contributions

The study was designed by RJ, KF, AP and JT. Analysis was performed by RB and KC. The first draft of the paper was written by RJ and all authors provided critical input and revisions.
